# Increasing water use efficiency along the C_3_ to C_4_ evolutionary pathway: a stomatal optimization perspective

**DOI:** 10.1093/jxb/eru205

**Published:** 2014-05-23

**Authors:** Danielle A. Way, Gabriel G. Katul, Stefano Manzoni, Giulia Vico

**Affiliations:** ^1^Department of Biology, Western University, London, ON, Canada; ^2^Nicholas School of the Environment, Duke University, Durham, NC, USA; ^3^Department of Civil and Environmental Engineering, Duke University, Durham, NC, USA; ^4^Department of Crop Production Ecology, Swedish University of Agricultural Sciences, Uppsala, Sweden; ^5^Department of Ecology, Swedish University of Agricultural Sciences, Uppsala, Sweden; ^6^Department of Physical Geography and Quaternary Geology, Stockholm University, Stockholm, Sweden.

**Keywords:** C_3_–C_4_ intermediates, C_3_ photosynthesis, leaf gas exchange, photosynthetic model, stomatal conductance, water use efficiency.

## Abstract

C_4_ photosynthesis evolved independently numerous times, probably in response to declining atmospheric CO_2_ concentrations, but also to high temperatures and aridity, which enhance water losses through transpiration. Here, the environmental factors controlling stomatal behaviour of leaf-level carbon and water exchange were examined across the evolutionary continuum from C_3_ to C_4_ photosynthesis at current (400 μmol mol^–1^) and low (280 μmol mol^–1^) atmospheric CO_2_ conditions. To this aim, a stomatal optimization model was further developed to describe the evolutionary continuum from C_3_ to C_4_ species within a unified framework. Data on C_3_, three categories of C_3_–C_4_ intermediates, and C_4_ *Flaveria* species were used to parameterize the stomatal model, including parameters for the marginal water use efficiency and the efficiency of the CO_2_-concentrating mechanism (or C_4_ pump); these two parameters are interpreted as traits reflecting the stomatal and photosynthetic adjustments during the C_3_ to C_4_ transformation. Neither the marginal water use efficiency nor the C_4_ pump strength changed significantly from C_3_ to early C_3_–C_4_ intermediate stages, but both traits significantly increased between early C_3_–C_4_ intermediates and the C_4_-like intermediates with an operational C_4_ cycle. At low CO_2_, net photosynthetic rates showed continuous increases from a C_3_ state, across the intermediates and towards C_4_ photosynthesis, but only C_4_-like intermediates and C_4_ species (with an operational C_4_ cycle) had higher water use efficiencies than C_3_ *Flaveria*. The results demonstrate that both the marginal water use efficiency and the C_4_ pump strength increase in C_4_ *Flaveria* to improve their photosynthesis and water use efficiency compared with C_3_ species. These findings emphasize that the advantage of the early intermediate stages is predominantly carbon based, not water related.

## Summary

Using a stomatal optimization model, an abrupt change is foundnd in the relationship between carbon and water fluxes along the evolutionary gradient from C_3_ to C_4_ photosynthetic types.

## Introduction

While only 3% of the world’s terrestrial plant species use the C_4_ photosynthetic pathway, C_4_ species are responsible for some 20% of global gross primary productivity (Sage *et al.*, 2012). The high productivity of C_4_ plants is due to their efficient photosynthetic physiology, which includes an additional yet spatially separated metabolic cycle, mediated by phospho*enol*pyruvate carboxylase (PEPCase), to the conventional C_3_ Calvin–Benson cycle. This additional cycle results in high CO_2_ concentrations around Rubisco, thus suppressing the enzyme’s oxygenase function and nearly eliminating photorespiration and the associated carbon and energetic costs.

C_4_ photosynthesis has evolved independently at least 66 times in lineages throughout the plant kingdom and, in some of these lines, there are intermediate species that are neither C_3_ nor fully C_4_ (Sage *et al.*, 2012). Phylogenetic analyses in some evolutionary groups where the full range of C_3_, C_3_–C_4_ intermediates, and C_4_ species are found (such as the genus *Flaveria*) have confirmed that C_3_ photosynthesis is the basal state and C_4_ photosynthesis is more derived; these analyses also place photosynthetic intermediate species as evolutionary intermediates to these two photosynthetic types (McKown *et al.*, 2005). The C_3_–C_4_ intermediates can be classified into three categories based on the degree to which they express C_4_ traits: Type I intermediates show refixation of photorespiratory CO_2_ by Rubisco in enlarged bundle sheath cells; Type II species have increased PEPCase activity and some C_4_ cycle function; and C_4_-like species have an operational C_4_ cycle, but have some residual Rubisco activity in their mesophyll cells (Edwards and Ku, 1987).

Despite extensive research, the role of environmental factors in driving the evolution of C_4_ photosynthetic traits continues to draw significant attention (e.g. Osborne and Sack, 2012; Griffiths *et al.*, 2013). Much work has focused on the importance of a drop in atmospheric CO_2_ concentrations (*c*_*a*_) from near 1000 ppm to ~400 ppm ~30 million years ago (mya), where the predominant benefit from a CO_2_-concentrating mechanism would have been enhanced net CO_2_ fixation rates through suppression of photorespiration (Ehleringer *et al.*, 1997; Sage, 2004; Christin *et al.*, 2011; Gowik *et al.*, 2011; Sage *et al.*, 2012). While low *c*_*a*_ increases photorespiration, the effect is even greater when combined with high temperatures: very low *c*_*a*_ conditions, such as those of the last glacial period (~180 μmol mol^–1^) (Lüthi *et al.*, 2008), may have selected for traits in some C3 species to favour the capture and reassimilation of respired and photorespired CO_2_ to offset this stress (Busch *et al.*, 2013), but the detrimental effects of low *c*_*a*_ conditions on C_3_ species are further exacerbated at warmer temperatures (Campbell *et al.*, 2005). The warm regions where C_4_ species evolved therefore probably stimulated photorespiration considerably, but they also drove a concomitant increase in transpiration demand (Taylor *et al.*, 2012). It has been known for decades that C_4_ plants are more water use efficient than C_3_ species under the same conditions (e.g. Rawson *et al.*, 1977; Morison and Gifford, 1983); a spate of recent work has highlighted the role of other environmental variables that, along with low *c*_*a*_, may have contributed to the rise of C_4_ photosynthesis, such as dry or saline conditions. These recent studies have emphasized the role of C_4_ photosynthesis in improving plant water status and preventing hydraulic failure in these environments (Osborne and Sack, 2012; Griffiths *et al.*, 2013).

Since C_4_ species can maintain high photosynthetic rates even when stomatal conductance is low compared with their C_3_ counterparts, it follows that C_4_ photosynthesis promotes higher water use efficiencies (WUEs) than are found in C_3_ species (e.g. Rawson *et al.*, 1977; Morison and Gifford, 1983; Monson, 1989; Huxman and Monson, 2003; Kocacinar *et al.*, 2008; Osborne and Sack, 2012). This pattern is apparent in both forms of WUE: instantaneous WUE (WUE_i_, defined as the leaf carbon gain from net photosynthesis, *A*_net_, per unit water lost via transpiration, *E*) and marginal WUE [$\lambda =\frac{\partial A_{net}}{\partial E};$ see Lloyd and Farquhar (1994); Vogan and Sage (2011); Manzoni *et al.* (2011); note that this definition of λ is consistent with that of Hari *et al.* (1986), but the inverse of the same symbol used by Cowan and Farquhar (1977)]. The marginal WUE λ can also be interpreted as the cost of losing water in carbon units. Thus, the higher λ of C_4_ species implies that water loss is more costly for the carbon balance with respect to C_3_ species, so that C_4_ species operate at a relatively low *E*, but at comparable or higher *A*_net_. This finding is consistent with C_4_ photosynthesis preventing hydraulic failure by means of a tight stomatal regulation of water loss (Osborne and Sack, 2012). Yet what controls the stomatal behaviour and WUE across the evolutionary continuum of C_3_ to C_4_ species remains a subject of research (Vogan and Sage, 2011; Way, 2012) and frames the scope of this work.

Recent experiments have shown that instead of a gradual improvement in WUE from C_3_ species, across the intermediate range and to a full C_4_ pathway, the increase in WUE resembles a threshold effect: Type I and II intermediates have WUEs on a par with C_3_ species, while C_4_-like species have a high WUE akin to C_4_ plants (Kocacinar *et al.*, 2008; Vogan and Sage, 2011). The development of the CO_2_-concentrating mechanism, which effectively pumps CO_2_ from the substomatal cavity into the chloroplasts where the Calvin–Benson cycle occurs, is thought to be the primary mechanism by which C_4_ plants enhance their WUE_i_. The present study therefore sought to investigate the connection between the evolutionary continuum of C_3_ to C_4_ photosynthesis and stomatal behaviour (which is a key factor in controlling WUE), by exploring the following questions.

(i) To what degree can stomatal optimization theories describe the WUE_i_ patterns in species that have photosynthetic characteristics intermediate between C_3_ and C_4_ species?
(ii) What are the relationships among the CO_2_-concentrating mechanism, λ, and WUE_i_ in these C_3_–C_4_ intermediates?

To address these questions, the genus *Flaveria* was used as a case study, since it contains species with C_3_ photosynthesis, all three intermediate photosynthetic types, and C_4_ photosynthesis (a system previously used by Huxman and Monson, 2003; Sage, 2004; McKown and Dengler, 2007; Kocacinar *et al.*, 2008; Gowik and Westhoff, 2011; Gowik *et al.*, 2011; Vogan and Sage, 2011; and others). Data on *Flaveria* are used to parameterize a stomatal optimization model and examine stomatal behaviour across the evolutionary range of C_3_ to C_4_ photosynthesis. By using a phylogenetically constrained system, the patterns of changes in the model parameters across the C_3_–C_4_ photosynthetic continuum can be simultaneously explored while minimizing evolutionary differences between groups that might otherwise confound the analysis.

## Theory

In the Farquhar *et al.* (1980) photosynthesis model, *A*_net_ is determined by the minimum of two limitations: the Rubisco carboxylation rate (*A*_C_) and ribulose-1,5-bisphosphate (RuBP) regeneration rate *A*_J_, and is commonly expressed as

$$A_{\text{net}}=\text{min}\left(A_{\text{C}},A_{\text{J}}\right)-R_{\text{d}},$$ (1)

where *R*_d_ is the daytime respiration rate (see Table 1 for symbols and definitions). Rubisco limitation occurs under saturating light or at low CO_2_ concentrations at the site of Rubisco, while RuBP regeneration tends to limit photosynthesis when *c*_*a*_ is high and light levels are low, resulting in a limited electron transport rate. The Rubisco-limited assimilation rate, *A*_C_, can be expressed as

**Table 1.** Symbols and their definitions used in the paper and model

Symbol	Definition	Units
*a*	Ratio of the molecular diffusivities of CO_2_ to water vapour	–
*A*_C_	Rubisco-limited CO_2_ assimilation rate	μmol m^–2^ s^–1^
*A*_J_	RuBP regeneration-limited net CO_2_ assimilation rate	μmol m^–2^ s^–1^
*A*_net_	Net CO_2_ assimilation rate	μmol m^–2^ s^–1^
*c*_a_	Atmospheric CO_2_ concentration	μmol mol^–1^
*c*_bs_	Bundle sheath CO_2_ concentration	μmol mol^–1^
*c*_c_	Chloroplastic CO_2_ concentration	μmol mol^–1^
CE	Carboxylation efficiency	mol m^–2^ s^–1^
*c*_i_	Intercellular CO_2_ concentration	µmol mol^–1^
*D*	Vapour pressure deficit	kPa
*E*	Transpiration rate	mmol m^–2^ s^–1^
*g*_s_	Stomatal conductance	mmol m^–2^ s^–1^
*J*	Electron transport rate	μmol m^–2^ s^–1^
*k*_1_	Maximum photosynthetic rate of the hyperbolic model (Equation 4)	μmol m^–2^ s^–1^
*k*_2_	Half-saturation constant for the hyperbolic model (Equation 4)	–
*K*_c_	Michaelis–Menten constant for Rubisco carboxylation	μmol mol^–1^
*K*_cair_	Half-saturated constant of the Rubisco-limited photosynthesis	μmol mol^–1^
*k*_cat_	Catalytic constant for Rubisco	mol mol^–1^ s^–1^
*K*_o_	Michaelis–Menten constant for Rubisco oxygenation	mmol mol^–11^
*O*	Atmospheric oxygen concentration	mmol mol^–1^
*Q*	Irradiance	μmol m^–2^ s^–1^
*q*	Temperature coefficient	–
*R*_d_	Day respiration	μmol m^–2^ s^–1^
*T*_l_	Leaf temperature	°C
*V*_c,ma*x*_	Maximum carboxylation rate of Rubisco	μmol m^–2^ s^–1^
*V*_c,max25_	Maximum carboxylation rate of Rubisco at 25 °C	μmol m^–2^ s^–1^
*V*_p_	PEPCase rate	μmol m^–2^ s^–1^
WUE	Water use efficiency	mmol mol^–1^
WUE_*i*_	Instantaneous water use efficiency	mmol mol^–1^
α	Kinetic constant (Equations 5 and 6)	–
α_p_	Leaf absorptivity	–
α_1,2_	Parameter groups (Equations 8–11)	–
β_1,2,3_	Parameter groups (Equations 8–11)	–
ε_m_	Maximum photochemical efficiency	–
γ	Parameter groups (Equations 8–11)	–
Γ	CO_2_ compensation point	μmol mol^–1^
Γ*	CO_2_ compensation point in the absence of mitochondrial respiration	μmol mol^–1^
η	C4 pump strength	–
λ	Marginal water use efficiency	mmol mol^–1^

$$A_{C}=V_{c,max}\frac{c_{c}-\Gamma^{\text{*}}}{c_{c}+K_{cair}},$$ (2)

where ***V***_c,max_ is the maximum Rubisco carboxylation rate, *c*_c_ is the CO_2_ concentration at the photosynthetic site, Γ* is the CO_2_ compensation point in the absence of mitochondrial respiration, and $K_{cair}=K_{c}\left(1+O/K_{o}\right),$ with *K*_c_ and *K*_o_ being the Michaelis–Menten constants for Rubisco CO_2_ fixation and oxygen inhibition, and *O* is the oxygen concentration in the air (21%). Conversely, the RuBP-limited assimilation rate is constrained by the rate of electron transport, *J*, and can be expressed as

$$A_{J}=\frac{J}{4}\frac{c_{c}-\Gamma^{\text{*}}}{c_{c}+2\Gamma^{\text{*}}},$$ (3)

where the electron transport rate is given by *J*=α_p_ε_m_ *Q*, and *Q* is the irradiance, α_p_ is the leaf absorptivity, and ε_m_ is the maximum photochemical efficiency (Genty *et al.*, 1989).

To avoid discontinuities in *A*_net_ due to an abrupt transition from one limitation to another, the minimum function in Equation 1 has often been replaced by a quadratic function, at the cost of introducing an additional curvature parameter. An alternative approach is to approximate Equation 1 by a hyperbolic function (Vico *et al.*, 2013),

$$A_{C,J}=k_{1}\frac{c_{c}-\Gamma^{\text{*}}}{c_{c}+k_{2}},$$ (4)

where $k_{1}=\frac{J}{4}\;\text{and}\;k_{2}=JK_{cair}/\left(4\;V_{c,max}\right)$ . Such a representation ensures that at low $c_{c}/k_{2},\;A_{C,J}\approx A_{C}$ and at large $c_{c}/k_{2},A_{C,J}\approx A_{J}.$ When $c_{c}/k_{2}$ is approximately unity, both Rubisco and RuBP regeneration rates exert comparable limitations on photosynthesis. Hereafter, this regime is referred to as the co-limitation regime. Under CO_2_-limited (or light-saturated) conditions in which k_1_=*V*_c,max_ and k_2_=*K*_cair_, the optimal solution is identical to the one obtained by Katul *et al.* (2010) for non-linear photosynthetic kinetics without light limitation. Based on Equation 4, net photosynthesis is obtained as *A*_net_=*A*_C,J_–*R*_d_.

Although there are numerous physiological and anatomical traits that underlie the development of the CO_2_-concentrating mechanism in C_4_ plants (e.g. McKown and Dengler, 2007; Sage *et al.*, 2012), for modelling purposes, the simplest description of the effect of such a pump is to assume that the CO_2_ concentration at the site where photosynthesis occurs is *c*_c_=η*c*_i_, where η represent the strength of the CO_2_-concentrating pump. The value of η encompasses not only the development of C_4_ biochemistry across the evolutionary gradient of species, but also biochemical and anatomical features that affect mesophyll conductance. In C_4_ species, η>1 (Manzoni *et al.*, 2011); while it is slightly smaller than unity in C_3_ species due to the need to diffuse CO_2_ through the mesophyll, the lack of specific data on mesophyll conductance meant this had to be neglected and η=1 was set for C_3_ species.

In the following, the pump strength, η, is estimated from the slope of the *A*_net_(*c*_i_) curve. Employing this simple description of the CO_2_-concentrating mechanism results in a simpler photosynthesis model than by considering explicitly PEPCase kinetics (Collatz *et al.*, 1992; Laisk and Edwards, 2000; von Caemmerer, 2000, 2013; Vico and Porporato, 2008), thereby allowing data sets collected across different experiments and conditions to be compared. Nevertheless, the parameter η can be linked to the kinetics of PEPCase. The CO_2_ concentration in the stomatal cavity (*c*_i_) is assumed to be transported by PEPCase activity and the shuttling of C_4_ acids to the bundle sheath (the site of photosynthesis), where the CO_2_ concentration reaches *c*_bs_. When PEPCase kinetics are assumed to be linear for illustration (but see von Caemmerer, 2000 for more detailed and non-linear models), then

$$V_{\text{P}}=\alpha c_{\text{i}},$$ (5)

where α is the kinetic constant of the process. Setting *V*_p_=*A*_net_ (from Equation 4) to guarantee continuity in the C fluxes from the stomatal cavity to the site of photosynthesis provides an equation to be solved for η, leading to

$$\eta =\frac{k_{1}\Gamma^{\text{*}}+k_{2}\left(\alpha c_{i}+R_{d}\right)}{c_{i}\left(k_{1}-\alpha c_{i}-R_{d}\right)}.$$ (6)

Equation 6 shows that the pump efficiency η in principle depends on the photosynthetic parameters as well as *c*_i_. However, neglecting *R*_d_ and assuming Γ*<<*c*_i_ and α*c*_i_<<*k*_1_, it can be shown that $\eta =\alpha k_{2}/k_{1},$ which is a constant at a given temperature and light level. Therefore, when respiration is small and photosynthetic capacity is large, a constant efficiency η captures the main effect of the PEPCase on the photosynthetic rate. Outside these simplifications, the assumption of a constant η can only be regarded as a first-order approximation.

The combination of the hyperbolic function in Equation 4 with the simplified description of the CO_2_ pumping mechanism based on η (i.e. *c*_c_=η*c*_i_) provides a tool to describe CO_2_ demand within a common framework valid across the C_3_ to C_4_ evolutionary continuum. Despite the inherent simplifications, this model is in good agreement with earlier, more complex photosynthesis models for C_3_–C_4_ intermediates and C_4_ species (von Caemmerer, 1989; Collatz *et al.*, 1992) (data not shown; for an example of model comparison for C_3_ species, see fig. 1 in Vico *et al.*, 2013), thus lending support to the present approach.

**Fig. 1.**
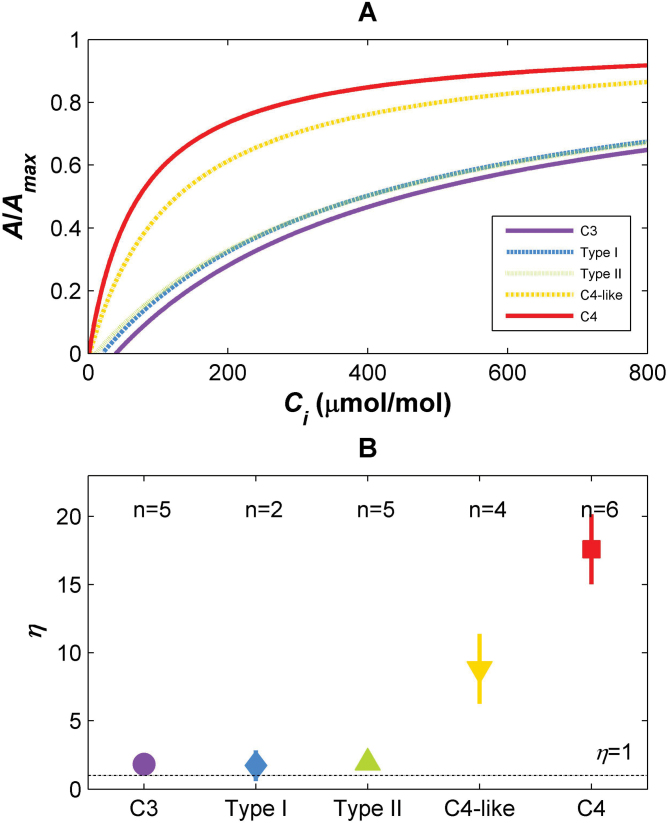
(A) Responses of net CO_2_ assimilation rates (*A*_net_) to increases in intercellular CO_2_ concentration (*c*_i_), relativized to maximum *A*_*net*_ (=*A*_max_) for each photosynthetic type, as commonly presented in the literature. (B) Estimated η for each photosynthetic type. Means ±SE, *n* indicated at the top, dotted line indicates η=1. C_3_ species, purple circles and solid line; Type I species, blue diamonds and dashed line; Type II species, green triangles and dotted line; C_4_-like species, yellow inverted triangles and dashed-dotted line; C_4_ species, red squares and solid line.

The biochemical demand for CO_2_ described by *A*_C,J_ is met by CO_2_ supplied by the atmosphere via Fickian diffusion at a rate given by

$$A_{\text{net}}=g_{\text{s}}\left(c_{\text{a}}-c_{\text{i}}\right),$$ (7)

where *g*_s_ is the stomatal conductance and *c*_a_ is the atmospheric CO_2_ concentration. For a given *c*_a_, set of environmental conditions (such as *Q* and temperature), and physiological properties determining *k*_1_ and *k*_2_, the atmospheric supply and biochemical demand for *A*_C,J_ constitute two equations with three unknowns: *g*_s_, *c*_a_, and *A*_net_. Hence, one additional equation is needed to close this system of equations mathematically.

This additional equation can take on the form of an optimality rule, whereby stomata are assumed to operate so as to maximize their carbon gain at a given water loss cost (Cowan and Farquhar, 1977; Hari *et al.*, 1986). This hypothesis is equivalent to maximizing a Hamiltonian function *H*=*A*_net_–λ*E*, where *E*=a*g*_s_ *D* is the leaf transpiration rate (assuming a perfectly coupled canopy), and a=1.6 is the ratio of the molecular diffusivities of CO_2_ to water vapour. Combining the biochemical demand with atmospheric supply so as to eliminate *c*_i_, and thereby expressing *A*_C,J_ as a function of *g*_s_, inserting the outcome into the Hamiltonian, and setting $\partial H/\partial g_{s}$ =0, leads to a quadratic equation in *g*_s_ (Vico *et al.*, 2013). Solving this equation for *g*_s_ results in a solution for optimal *g*_s_ as a function of biochemical parameters (η, *V*_c,max_, *K*_cair_, *R*_d_, and Γ*), environmental conditions (*c*_a_ and *D*), and the optimization parameter λ. The explicit functional form for optimal stomatal conductance is determined from the solution to the optimality problem as

$$\frac{\partial H}{\partial g_{s}}=\frac{\partial (A_{net}-\lambda E)}{\partial g_{s}}=0\overset{\text{optimality condition}}{\to }g_{s}=\frac{\beta_{1}+\sqrt{\beta_{2}}}{\beta_{3}}$$ (8)

where

$$\beta_{1}=-\gamma \eta \left(k_{2}+\alpha_{2}\right)\left[k_{1}\left(k_{2}-\eta c_{\text{a}}+2\Gamma^{\text{*}}\right)+\alpha_{1}R_{\text{d}}\right],$$ (9)

$$\begin{array}{l} \beta_{2}=-\gamma \eta k_{1}\left(k_{2}+\Gamma^{\text{*}}\right)\left(k_{2}+\alpha_{2}\right)\\ \;{\left[k_{2}+\eta \left(c_{\text{a}}-2\gamma \right)\right]}^{2}\left[k_{1}\left(\Gamma^{\text{*}}-\eta c_{\text{a}}\right)+\alpha_{1}R_{\text{d}}\right], \end{array}$$ (10)

$$\beta_{3}=\gamma \alpha_{1}^{2}\left(k_{2}+\alpha_{2}\right)$$ (11)

In Equations 9, 10, and 11, α_1_=*k*_2_+η*c*_a_, α_2_=η(*c*_a_–γ), and γ=αλ*D*. Therefore, the optimal stomatal conductance depends on λ, which by using the optimization condition $\partial H/\partial g_{s}$ =0 can be shown to be equal to the definition of the marginal WUE, i.e.

$$\lambda =\frac{\partial A_{net}}{\partial E}=\frac{\partial A_{net}}{\partial g_{s}}{\left(\frac{\partial E}{\partial g_{s}}\right)}^{-1}.$$ (12)

Equation 12 provides a physical interpretation for λ, but does not give additional information (the optimization condition has already been used in Equation 8). Hence, λ needs to be determined to close the optimization problem mathematically. Although λ changes as a function of time when soil moisture declines during a dry period (Manzoni *et al.*, 2013), under well-watered conditions or stable moisture levels, λ can be considered time-invariant.

Before applying the proposed model, it is important to summarize its key assumptions and simplifications:

(i) Photosynthetic kinetics are described by a hyperbolic function of *c*_i_ bridging a CO_2_-limited regime (where *A*_net_ scales linearly with *c*_i_) and a light-limited regime (where *A*_net_ depends solely on a light level).
(ii) PEPCase kinetics are described by a single efficiency parameter η, which approximates more complex models well (Collatz *et al.*, 1992; von Caemmerer, 2000) when respiration terms are small. Also mesophyll resistance is neglected, due to a lack of data across these species; this assumption implies that the estimated η could be inflated under dry conditions (though these are not the conditions considered when inferring the marginal WUE).
(iii) Stomatal conductance is obtained from an optimization argument assuming that the marginal WUE is constant—a reasonable approximation for experiments under controlled conditions and stable moisture levels (Manzoni *et al.*, 2013). Thus, λ is used as a fitting parameter affecting the stomatal conductance in Equation 8.

Clearly, these assumptions could be relaxed, thereby improving realism. However, relaxing these assumptions reduces the ease of interpretation of the derived equations and the ability to compare across a wide range of data sets due to more required parameters. Once the optimal *g*_s_ is determined, *A*_C,J_, *E*, and *c*_i_ can then be computed. This model allows the quantification of *A*_net_ and *g*_s_ for C_3_, C_4_, and C_3_–C_4_ intermediate species within a common framework and as a function of both environmental conditions (air temperature, *Q*, *D*, and *c*_a_) and species-specific parameters (η, λ, *V*_c,max_, *K*_cair_, and Γ*)). As such, after showing that the modelled response of *A*_net_ to changes in *g*_s_ is well captured assuming optimal stomatal behaviour, the model is used to investigate how *A*_net_ and WUE_i_ are altered by changes in η, λ, and *c*_a_, thus following the steps of the hypothesized evolution of C_3_–C_4_ intermediates and C_4_ species from C_3_ plants.

## Data availability and model parameterization

To examine the consequences of intermediacy on stomatal behaviour, WUE_i_, and λ, *Flaveria* species were used where gas exchange measurements for C_3_, C_4_, and intermediate species have been previously characterized. The data included C_3_ species (*F. cronquistii* and *F. pringleii*), Type I intermediates (*F. angustifolia*, *F. chloraefolia*, *F. pubescens* and *F. sonorensis*), Type II intermediates (*F. floridana* and *F. ramosissima*), C_4_-like intermediates (*F. brownii*, *F. palmeri* and *F. vaginata*), and C_4_ species (*F. australasica*, *F. bidentis*, *F. kochiana* and *F. trinervia*) (see Supplementary Table S1 available at *JXB* online). The most recent photosynthetic classification of the species (Vogan and Sage, 2011) was employed. Responses of *g*_*s*_ and *A*_*net*_ to variation in *c*_*a*_, and responses of *A*_net_ to changes in *g*_*s*_ were either taken from the literature or digitized from published graphs (Monson, 1989; Vogan and Sage, 2011). Environmental conditions (*Q*, *D*, *c*_*a*_, and leaf temperature) in model runs were matched to the measurement conditions described for the experimental data. The range in λ necessary to capture measured responses in gas exchange was explored in *Flaveria* species from all photosynthetic types.

To parameterize the above model for *Flaveria*, *V*_c,max_ values were derived for Rubisco from 15 *Flaveria* species that spanned C_3_ to C_4_ photosynthetic types using *in vitro* measurements of catalytic constants (or turnover numbers, *k*_cat_) (Kubien *et al.*, 2008) and Rubisco site concentrations from the same experiment (D. Kubien, personal communication) (Table 2). The Michaelis–Menten constants *K*_c_ and *K*_o_ for Rubisco were also taken for each *Flaveria* species from Kubien *et al.* (2008). Rubisco kinetics were adjusted to 30 °C to match conditions in the carboxylation efficiency studies using correction equations and coefficients from Campbell and Norman (1998) (Table 2). *K*_c_ and *K*_o_ were temperature adjusted by multiplying their values at 25 °C by exp[*q*(*T*_l_–25)], where *q* is the temperature coefficient for that parameter (0.074 for *K*_c_ and 0.015 for *K*_o_) and *T*_l_ is leaf temperature. *V*_c,max_ was adjusted as $V_{c,max}=\frac{V_{c,max25}\text{exp}[0.088\left(T_{l}-25\right)]}{1+\text{exp}[0.29\left(T_{l}-41\right)]}$ , where *V*_c,max25_ is the maximum carboxylation rate at 25 °C. The Γ* values for each of the five photosynthetic types were approximated using averaged values of Γ (the CO_2_ compensation point) from *Flaveria* species in Ku *et al.* (1991), assuming that day respiration of mitochondria is small (*R*_d_=0.015*V*_c,max_) and can be ignored (Table 2). Because light conditions varied across experiments, the estimation of *J*(α_p_ε_m_ *Q*) needed in determining $a_{1}=J\;\text{and}\;a_{2}=Jk_{2}/\left(4\;V_{c,max}\right)$ requires an estimate of the product α_p_ε_m_ (not their individual values). The value for α_p_ was set at 0.8 (based on values for C_3_ and C_4_ species in von Caemmerer, 2000 and Collatz *et al.*, 1992, respectively), ε_m_ was 0.1mol mol^–1^ (similar to Norman and Campbell, 1998; Cheng *et al.*, 2001; Taiz and Zeiger, 2010), and *Q* was set for the irradiance used in individual papers being modelled. Hence, α_p_ε_m_=0.08, resulting in α_1_=0.08*Q* when RuBP regeneration limits photosynthesis. This estimate is consistent with conventional values for C_3_ species (Campbell and Norman, 1998; see table 14.1) though α_p_ε_m_ may be more uncertain for C_4_ species and C_3_–C_4_ intermediates. For C_3_–C_4_ intermediates, Monson (1989) and Monson and Jaeger (1991) report mid-day photosynthetic rates for several species including *F. floridana* between 15 μmol m^–2^ s^–1^ and 45 μmol m^–2^ s^–1^ at light levels ranging from *Q*=1500 μmol m^–2^ s^–1^ to 2000 μmol m^–2^ s^–1^. Because $A_{J}\approx J/4$ (assuming that Γ* is negligible in Equation 3), it follows that the measured *A* are consistent with the estimate *J*=0.08*Q*, which gives an *A*_net_ of 40 μmol m^–2^ s^–1^. This evidence supports the assumption that α_p_ε_m_ is stable across photosynthetic types. A more rigorous parameterization would require direct observations of α_p_ε_m_ or reliable *V*_c,max_–*J*_max_ relationships across the C_3_–C_4_ continuum.

**Table 2.** Parameter values (based on mean values from experimental data corrected to 30 °C) used for modelling photosynthesis for each photosynthetic type of Flaveria

	CE (mol m^–2^ s^–1^)	Γ (μol mol^–1^)	*V*_c,max_ (μmol m^–2^ s^–1^)	*K*_c_ (μmol mol^–1^)	*K*_o_ (mmol mol^–1^)	*R*_d_ (μmol m^–2^ s^–1^)	λ (mmol mol^–1^)	η (unitless)
References for values	Sudderth *et al.* (2007), citing Dai *et al.* (1996)	Ku *et al.* (1991)	Kubien *et al.* (2008) (and pers. comm.)	Kubien *et al.* (2008)	Kubien *et al.* (2008)	Estimated as 0.015 *V*_c,max_		
C_3_ species	0.11	61.36	53.20	494.9	575.8	0.8	0.826	1.83
Type I	0.079	25.45	73.40	516.6	665.7	1.10	0.739	1.73
Type II	0.13	9.20	66.72	547.1	614.6	1.00	0.754	1.93
C_4_-like	0.27	4.93	34.10	690.3	422.3	0.51	3.267	8.83
C_4_ species	0.47	3.32	39.52	898.7	1631.8	0.59	3.410	17.60

CE, carboxylation efficiency; Γ, CO_2_ compensation point; *V*_c,max_, *in vitro* maximum carboxylation rate of Rubisco; *K*_c_ and *K*_o_ (at 30 °C), Rubisco Michaelis–Menten constants for CO_2_ and O_2_, respectively; *R*_d_, day respiration rates; λ, marginal water use efficiency; η, C_4_ pump strength.

**Fig. 2.**
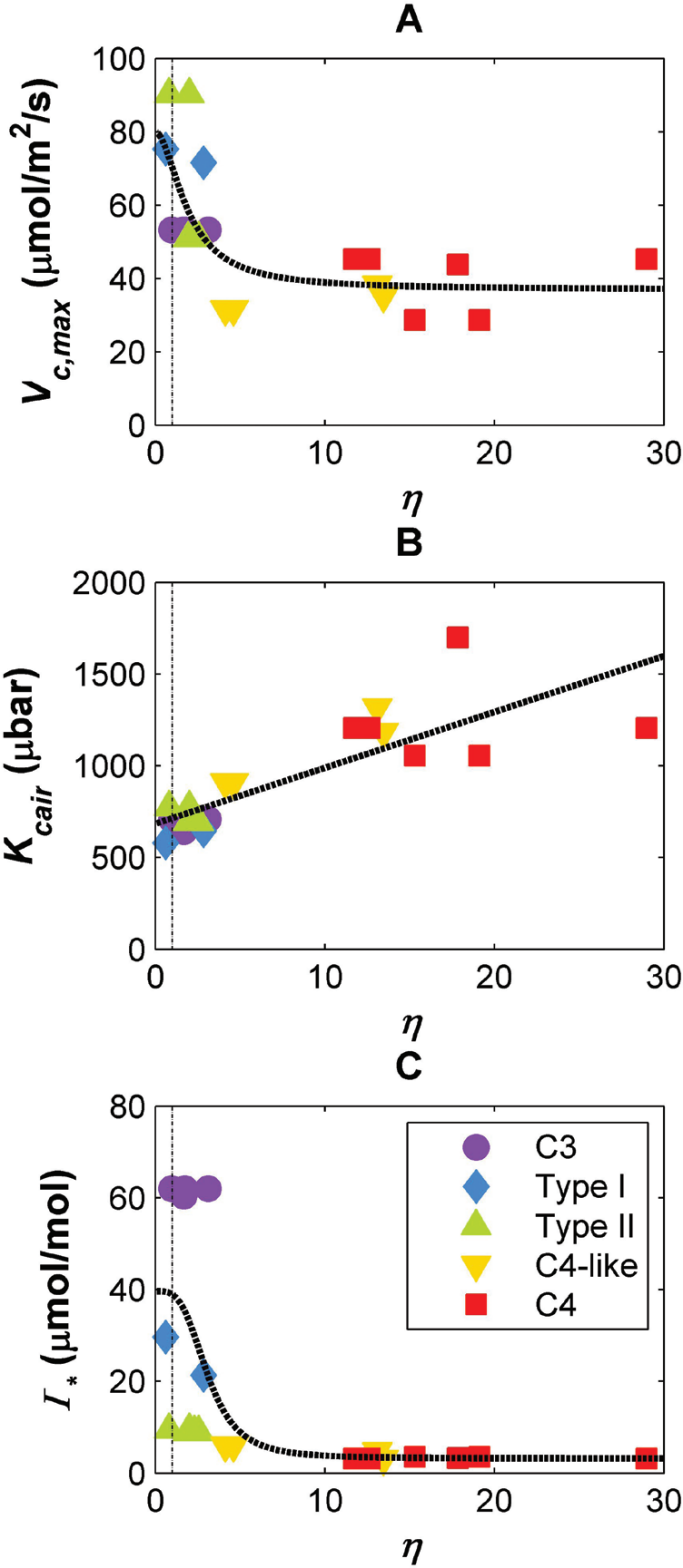
Relationships between the pump strength of the CO_2_-concentrating mechanism (η) and: (A) maximum carboxylation capacity of Rubisco (*V*_c,max_); (B) $K_{cair}=K_{c}\left(1+O/K_{o}\right)$ ; and (C) the CO_2_ compensation point in the absence of mitochondrial respiration (Γ*) for C_3_, C_4_, and C_3_–C_4_ intermediate species. Solid lines are fit to data; vertical dotted lines indicate η=1. C_3_ species, purple circles; Type I species, blue diamonds; Type II species, green triangles; C_4_-like species, yellow inverted triangles; C_4_ species, red squares.

Data are taken from literature sources as outlined in the text (see also species-specific data points in Fig. 2 and Supplementary Table S1 at JXB online); λ and η are calculated values.

Carboxylation efficiencies [CEs; i.e, the initial slope of the *A*_net_(*c*_i_) curve] measured under saturating light for *Flaveria* species were taken from Krall *et al.* (1991) and Sudderth *et al.* (2007), citing Dai *et al.* (1996). Based on Equation 2, at low *c*_i_ the slope of the *A*_net_(*c*_i_) is approximately $\text{CE}=V_{c,max}\eta /K_{cair}$ , so that $\eta =\text{CE}K_{cair}/V_{c,max}.$ Therefore, knowledge of Γ, *K*_cair_, *K*_c_, and *K*_o_ allowed an estimate of η for each *Flaveria* species.

Finally, the Vogan and Sage (2011) gas exchange data set was used to infer how λ changes across the evolutionary pathway from C_3_ to C_4_ species. In that study, a range of *g*_s_ and *A*_net_ values was obtained by altering the nitrogen availability for individuals of all photosynthetic types considered, while water was amply supplied, so λ can be considered time-invariant. As a consequence of different nutrient availability, a range of photosynthetic capacities and respiration rates were obtained. Since there is no way of knowing these biochemical parameters, a simplified but more robust approach to estimate λ was adopted that only requires gas exchange rates and photosynthetic type-averaged Γ and η (assuming λ is substantially unaltered by nutrient availability). For this step, instead of using the definition (Equation 12), which requires knowledge of all the photosynthetic parameters, the stomatal optimization model was simplified by selecting light-saturated conditions, so that *R*_d_≈0 and the photosynthesis model is approximately linear. Following these simplifications, it can be shown that $A_{net}=g_{s}\sqrt{aD\lambda \left(c_{a}-\Gamma_{*}/\eta \right)}$ (Manzoni *et al.*, 2011), which allows estimating λ through a linear least square regression of *A*_net_ versus *g*_s_ constrained through the origin for each photosynthetic type. In previous works on different species, this approach to estimate λ was compared with results obtained without these simplifications. Such comparison showed that the differences between the two approaches was rarely more than 20% across a wide range of environmental and physiological conditions (see fig. 4 in Katul *et al.*, 2010), which is in the range of experimental variability [e.g. a mean standard deviation of 16% in light-saturated *A*_net_ estimates across individuals in a range of C_3_, C_3_–C_4_ intermediates, and C_4_ species (Vogan *et al.*, 2007)].

Gas exchange rates were also simulated under altered atmospheric CO_2_ concentrations. In this analysis, all biochemical parameters were maintained constant, but the possibility was considered that the marginal WUE increases linearly with CO_2_ concentrations (Manzoni *et al.*, 2011). Simulations with constant λ estimated as described above were thus compared with simulations with λ(*c*_a_)=λ_400_(*c*_a_/400). Including CO_2_ effects allows the robustness of the results to changes in λ to be tested.

## Results and Discussion

Recent work has stimulated new interest in the role that transpiration demands may have played in the evolution of C_4_ photosynthesis and traits associated with the C_4_ syndrome (Taylor *et al.*, 2011, 2012; Osborne and Sack, 2012; Griffiths *et al.*, 2013). These studies have emphasized that C_4_ photosynthesis not only benefits the carbon economy of a plant, but also has important implications for hydraulic traits, drought tolerance, and water use patterns, benefits that are maintained or enhanced when C_4_ plants are exposed to the low *c*_*a*_ conditions where C_4_ photosynthesis evolved (Ripley *et al.*, 2013). Here, λ is used as an ‘index’ of the cost of losing water in terms of carbon, and its variation along the evolutionary gradient from C_3_ to C_4_ photosynthesis is investigated.

Combining a stomatal optimization approach with measured biochemical parameters, realistic mean *A*_net_(*c*_i_) curves for each photosynthetic pathway in *Flaveria* were computed (Fig. 1A; Table 2). The corresponding estimated η values are reported in Fig. 1B. In the optimality model, recall that the parameter η represents an overall pump strength for the carbon-concentrating mechanism, which might naively have been expected to increase gradually from C_3_ towards C_4_ plants. Instead, the analysis here suggests that η was relatively stable and similar to that for C_3_ species (η=1 or slightly above 1 due to unavoidable errors in the estimation) until reaching C_4_-like species. The relatively constant η between C_3_, Type I, and Type II *Flaveria* species occurred despite there being an increase in the initial slope of the *A*_net_(*c*_i_) curve (e.g. the carboxylation efficiency) across these groups. Instead of being attributed to η, the steeper initial slopes in the Type I and Type II intermediates in comparison with the C_3_ species were caused by higher *V*_c,max_ values for Rubisco based on *in vitro* assays of the enzyme kinetic parameters (Table 2; Fig. 2A), consistent with positive selection on Rubisco across the C_3_ to C_4_ gradient (Kapralov *et al.*, 2011). Thus, there was no increase in η until the C_4_-like species were reached; at this point, η values were about half-way between those of the full C_3_ and C_4_ photosynthetic groups. The greater variation in η estimates in species closer to the C_4_ end of the spectrum is therefore probably due to the greater range of pump strengths possible as the carbon-concentrating mechanism is established and to the species-level diversity in *V*_c,max_ values (Supplementary Table S1 at *JXB* online). While there were sharp changes in *in vitro* Rubisco *V*_c,max_ between C_3_ species and the Type I and Type II intermediates, the change in the *K*_cair_ of Rubisco across the photosynthetic groups was more gradual until reaching the C_4_-like species (Fig. 2B), implying that these enzyme kinetic traits are not necessarily linked. The Γ* dropped sharply as η increased slightly above a value of 1 and then flattened (Fig. 2C).

**Fig. 3.**
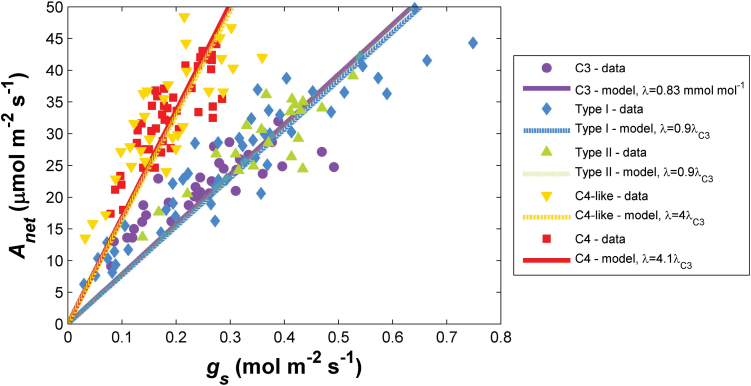
Relationships between stomatal conductance to CO_2_ (*g*_s_) and net CO_2_ assimilation rate (*A*_net_) in *Flaveria* species across the C_3_ to C_4_ photosynthetic range. Data points are from Vogan and Sage (2011); lines are obtained by analytical least-square fitting of the water use efficiency λ, employing a linearized version of the stomatal optimization model (Manzoni *et al.*, 2011) for analytical tractability.

While it might, *a priori*, seem reasonable to expect a steady increase in WUE_i_ from C_3_ species through the intermediate *Flaveria* species and to full C_4_ plants, this was not borne out by the data, in agreement with published findings. Huxman and Monson (2003) showed that the WUE_i_s of C_3_–C_4_ intermediates were similar to C_3_ WUE_i_ values, while C_4_ WUE_i_ values were considerably higher. Vogan and Sage (2011) also found no evidence for a gradual transition in the slope between *A*_net_ and *g*_*s*_ in C_3_–C_4_ intermediates, but rather a sharp increase between Type II intermediates and C_4_-like intermediates. [Note that WUE_i_ is a proxy for λ if a linear *A*_net_(*c*_i_) curve is assumed.] In our re-analysis of the Vogan and Sage (2011) data set, the reported gas exchange data could be readily described with the optimality model for C_3_, and Type I and Type II intermediates using the estimated changes in η (as in Fig. 1B), but without significant changes in λ across photosynthetic pathways (Fig. 3). This result implies that there is little change in the relationship between carbon and water from that of a C_3_ species in these early intermediate steps. However, in C_4_-like intermediates, a strong C_4_ pump (i.e. η=8) was accompanied by a quadrupling of λ compared with that used to characterize the data for Type I and II intermediates. Thus, the stomatal optimization approach could be successfully used to capture key changes in the measured relationship between *A*_net_ and *g*_s_ across the C_3_–C_4_ spectrum using the estimated η values, but only when the marginal WUE of the C_4_-like and C_4_ species was modelled to be 4-fold greater than that of the C_3_ species (Fig. 3). This increase in λ unambiguously indicates a higher carbon cost for losing water in the C_4_-like and C_4_ species. In the optimization model, the long-term *c*_*i*_/*c*_a_ dictates λ, because λ∞(1–*c*_i_/*c*_a_)^2^. Therefore, the increase in λ is generated by a decline in *c*_*i*_ (where *c*_*a*_ is assumed to be 400 μmol mol^–1^). This finding suggests that the increase in C_4_ WUE_i_ values, the increase in λ, and the decline in *c*_i_ (as expected by the presence of a C_4_ pump) are all interconnected and predicted from the proposed stomatal optimization model.

**Fig. 4.**
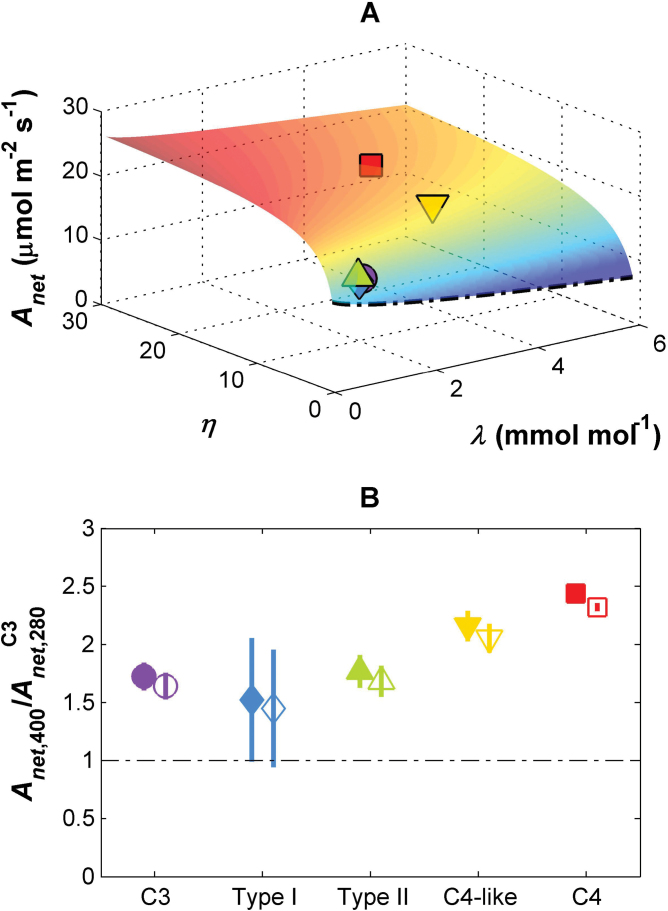
(A) Modelled relationships between net CO_2_ assimilation rate (*A*_net_), marginal water use efficiency (λ), and the CO_2_-concentrating pump strength (η) modelled at current CO_2_ concentrations (400 μmol mol^–1^); *V*_c,max_, *K*_cair_, and Γ* vary with η according to the relationships in Fig. 2; vapour pressure deficit (*D*) was set to 1.5 kPa, leaf temperature to 30 °C, *Q* to 1500 μmol m^–2^ s^–1^. Mean values of λ and η for each of the five photosynthetic types are indicated on the surface. (B) The ratio of *A*_*net*_ at current atmospheric CO_2_ levels versus *A*_net_ of C_3_ *Flaveria* at low atmospheric CO_2_ concentrations (280 μmol mol^–1^) (*A*_net_,_400_/*A*_net_^C3^,_280_) for each photosynthetic group; means ±SE across species; filled symbols refer to constant λ, open symbols to λ increasing linearly with *c*_*a*_; the dashed-dotted line indicates a ratio of 1. C_3_ species, purple circle; Type I species, blue diamond; Type II species, green triangle; C_4_-like species, yellow inverted triangle; C_4_ species, red square.

**Fig. 5.**
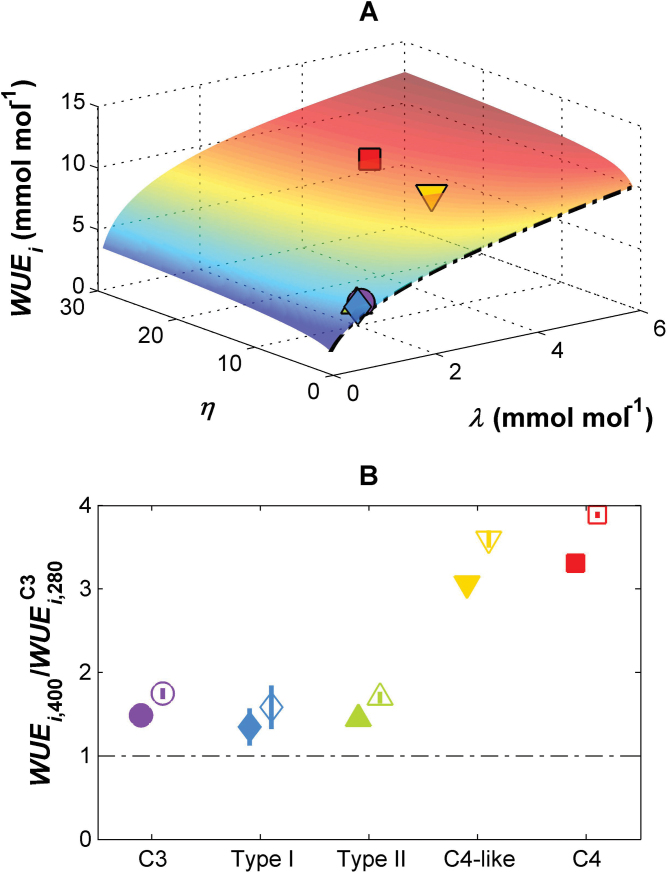
Modelled relationships between instantaneous water use efficiency (WUE_i_, the ratio of *A*_net_ to *E*), marginal WUE (λ), and the CO_2_-concentrating pump strength (η) modelled at current CO_2_ concentrations (400 μmol mol^–1^); *V*_c,max_, *K*_cair_, and Γ* vary with η according to the relationships in Fig. 2; vapour pressure deficit (*D*) was set to 1.5 kPa, leaf temperature to 30 °C, *Q* to 1500 μmol m^–2^ s^–1^. Mean values of λ and η for each of the five photosynthetic types are indicated on the surface. (B) The ratio of WUE_i_ at current atmospheric CO_2_ levels versus the WUE_i_ of a C_3_ *Flaveria* at low atmospheric CO_2_ concentrations (280 μmol mol^–1^) (WUE_i400_/WUE^C3^_i280_) for each photosynthetic group; means ±SE across species; filled symbols refer to constant λ, open symbols to λ increasing linearly with *c*_*a*_; the dashed-dotted line indicates a ratio of 1. C_3_ species, purple circle; Type I species, blue diamond; Type II species, green triangle; C_4_-like species, yellow inverted triangle; C_4_ species, red square.

Since C_4_ photosynthesis evolved under low *c*_a_, with the transition in *Flaveria* occurring within the last 3 million years (Sage *et al.*, 2012), the effect of varying η and λ on *A*_net_ and WUE_i_ was further investigated under both current (400 μmol mol^–1^) and low CO_2_ concentrations (280 μmol mol^–1^; Figs 4, 5), allowing *V*_c,max_, *K*_cair_, and Γ* to vary along with η as per the relationships in Figs 1 and 2, but keeping λ constant. In the results from both current and low *c*_a_ levels, increases in η initially induce a sharp increase in *A*_net_, as more CO_2_ is concentrated around Rubisco, with a diminishing response above a certain η (η=10 for 400 μmol mol^–1^ CO_2_; Fig. 4A). Moreover, higher values of λ decrease *A*_net_ in both environments, so that the slight changes in η and λ in Type I and II species compared with C_3_ plants generate a similar *A*_net_ in the three groups (Fig. 4A). Increased *c*_a_ (from 280 μmol m^–2^ s^–1^ to 400 μmol mol^–1^) elevated *A*_net_ in C_3_ species by 80% due to greater substrate availability (Fig. 4B). Compared with a C_3_ *Flaveria* at these low *c*_a_, C_3_–C_4_ intermediates also have higher *A*_net_ in modern CO_2_ concentrations, with a gradual increase in the stimulation of *A*_net_ with respect to C_3_ values (Fig. 4B). In contrast to the *A*_net_ results, increases in η have little impact on WUE_i_ when λ is small, namely from C_3_ species to Type I or II species (shown for 400 μmol mol^–1^ CO_2_ in Fig. 5A). Moreover, while increases in *c*_a_ have increased WUE_i_ of C_3_ species by 30%, there is no gradual rise in WUE_i_ across the gradient of photosynthetic types, as there was with *A*_net_ (Fig. 5B). Instead, compared with a C_3_ *Flaveria* at 280 μmol mol^–1^ CO_2_, Type I and Type II intermediates have a similar 30% stimulation in WUE_i_, while C_4_-like and C_4_ species show a more than tripling of their WUE_i_ stimulation at modern CO_2_ levels (Fig. 5B).

If the marginal WUE is assumed to increase with atmospheric CO_2_ (e.g. Katul *et al.*, 2010; Manzoni *et al.*, 2011), the predicted *g*_s_ at *c*_*a*_=280 μmol mol^–1^ increases. As a consequence, photosynthesis also increases and the ratios of net photosynthesis at *c*_a_=400 μmol m^–2^ s^–1^ and 280 μmol mol^–1^ therefore decrease (Fig. 4B). Because the positive effect of changes in λ is larger on transpiration than on net photosynthesis, the WUE_i_ at the lower *c*_a_ decreases. As a result, the ratio of WUE_i_ at current and low CO_2_ concentrations is higher than when assuming a constant λ (Fig. 5B).

**Fig. 6.**
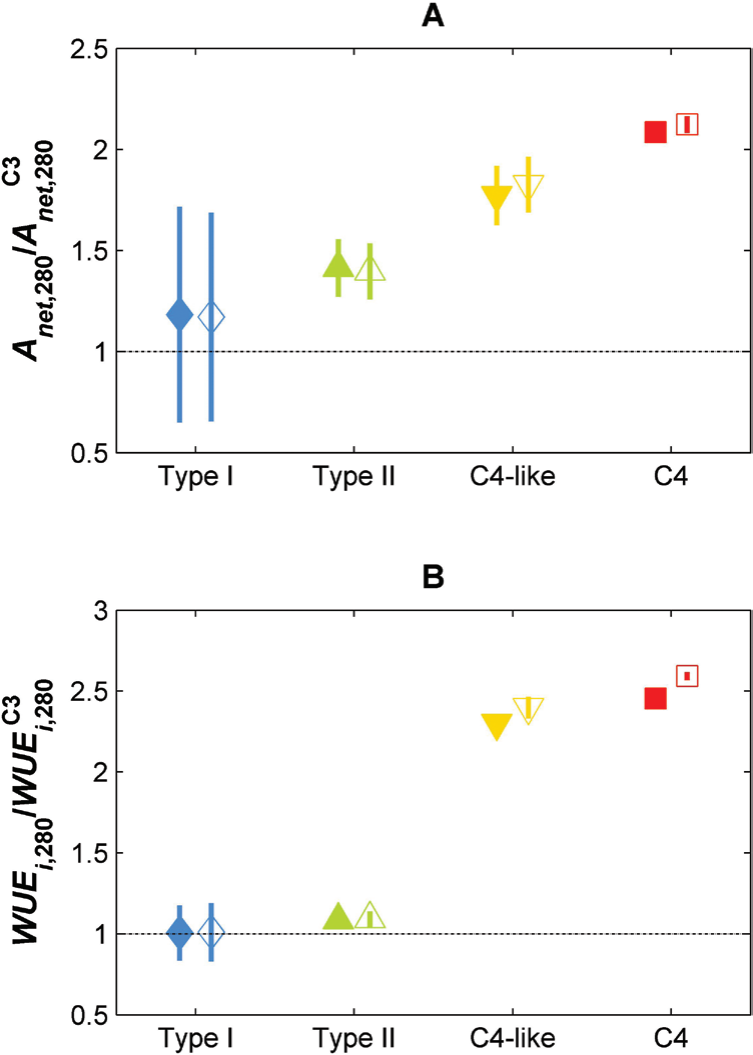
Comparison of (A) modelled photosynthetic rates and (B) modelled WUE_i_ among *Flaveria* species with different photosynthetic types at *c*_a_=280 μmol mol^–1^, expressed as ratios over the mean *A*_net_ and WUE_i_ for C_3_ species at *c*_*a*_=280 μmol mol^–1^. Symbols represent means ±SE across species (for fixed C_3_ *A*_net_ and WUE_i_ values); filled symbols refer to constant λ, open symbols to λ increasing linearly with *c*_*a*_; the dashed-dotted line indicates a ratio of 1. Other parameters are as in Figs 4 and 5.

The modelled changes in leaf-level performance between photosynthetic groups under low *c*_a_ are shown in Fig. 6. This figure quantifies the advantages of the intermediate and C_4_ species over the basal C_3_ state. At low *c*_a_, the estimated changes in η and λ for intermediate species provide a continuous, smooth gradient of increasing carbon gain, over a C_3_ *Flaveria* species (Fig. 6A). This trend is robust to changes in the λ(*c*_a_) relationship, as indicated by minor differences between filled and open symbols. A Type I intermediate has a 15% higher *A*_net_ than a C_3_ species, which could provide a competitive edge to the intermediate in a low CO_2_ environment; a similar jump in *A*_net_ is seen for each photosynthetic group along the evolutionary trajectory, in agreement with a recently proposed smoothly increasing fitness landscape for C_4_ evolution (Heckmann *et al.*, 2013). However, the same pattern is not apparent in the WUE_i_ results (Fig. 6B). There is no difference in the WUE_i_ estimated at 280 μmol mol^–1^ CO_2_ between C_3_, Type I and Type II *Flaveria* species. Instead, significant increases in WUE_i_ are only achieved in C_4_-like and C_4_ species, implying that the driving force for the initial steps towards C_4_ photosynthesis in this group was carbon based and not related to increasing WUE_i_.

Many of the features considered to pre-adapt a group to evolve C_4_ photosynthesis are related to leaf hydraulics, including increased vein density and enlarged bundle sheath cell size (McKown *et al.*, 2005; McKown and Dengler, 2007; Osborne and Sack, 2012; Sage *et al.*, 2012; Griffiths *et al.*, 2013). Stomatal anatomy also evolves along the transition from C_3_ to C_4_ photosynthesis, with C_4_ species having lower maximum stomatal conductance (due to either lower stomatal density or smaller stomatal size) than C_3_ congeners (Taylor *et al.*, 2012). While changes in whole-plant physiology are outside the scope of this work, these findings have stimulated interest in the role of plant water relations in the evolution of C_4_ photosynthesis. The results here indicate that while there is a gradual increase in carbon gain across the range from C_3_ to C_4_, there is no corresponding transition in either WUE_i_ or λ. Rather, increases in leaf-level WUE_i_ are only seen between Type II intermediacy and C_4_-like species (as noted by Kocacinar *et al.*, 2008; Vogan and Sage, 2011). However, this transition is accompanied by a rise in λ, indicating that a higher carbon cost is being incurred for water loss in C_4_-like and C_4_ species than in C_3_, or Type I or Type II intermediate species of *Flaveria*. This corresponds to the coordinated set of changes to the hydraulic architecture of *Flaveria* species, including lower leaf specific hydraulic conductivity and greater cavitation resistance in C_4_ and C_4_-like than C_3_ species (Kocacinar *et al.*, 2008), emphasizing the importance of the transition from having a functional C_4_ cycle for both the carbon and water economies of the plant.

## Conclusions

Using a stomatal optimization approach, the full range of C_3_, C_3_–C_4_ intermediates. and C_4_ gas exchange could be realistically modelled with the addition of a C_4_ pump strength parameter η, describing the effects of the C_4_ carbon-concentrating mechanism. The results here showed that, to capture the patterns apparent in measured gas exchange data, the carbon-based cost of losing water (λ) between C_3_, and Type I and Type II intermediates could be maintained constant, but λ had to be quadrupled to model C_4_-like and C_4_ *Flaveria* (at least within the confines of the optimality assumption of stomata). When leaf-level fluxes were modelled at low CO_2_, there was no evidence for a greater WUE_i_ in the C_3_–C_4_ intermediates (compared with a C_3_ *Flaveria*) until they developed a full C_4_ cycle. However, the model results suggest a steady increase in net carbon fixation rates across the C_3_ to C_4_ photosynthetic range. While this implies that carbon, not water, was the main driving pressure for the early steps of C_4_ evolution in this genus, the increase in λ indicates that there was a fundamental shift over the evolution of C_4_ photosynthesis between the relative costs of carbon and water, resulting in higher carbon costs of water losses.

## Supplementary data

Supplementary data are available at *JXB* online.

Table S1. Parameter table.

Supplementary Data
