# Supplementary material for: Increasing water use efficiency along the C3 to C4 evolutionary pathway: a stomatal optimization perspective
Source: J Exp Bot. 2014 May 23;65(13):3683–93. doi: 10.1093/jxb/eru205 (PMC4085968; doi:10.1093/jxb/eru205)
Supplement: Supplementary Data [file supp_65_13_3683__index.html]

Increasing water use efficiency along the C3 to C4 evolutionary pathway: a stomatal optimization perspective — Increasing water use efficiency along the C3 to C4 evolutionary pathway: a stomatal optimization perspective — Supplementary Data 

# Increasing water use efficiency along the C3 to C4 evolutionary pathway: a stomatal optimization perspective

## Supplementary Data

Data files

**Files in this Data Supplement:**

- Supplementary Data - Supplementary Data
