# Supplementary material for: Increasing water use efficiency along the C3 to C4 evolutionary pathway: a stomatal optimization perspective
Source: J Exp Bot. 2014 May 23;65(13):3683–93. doi: 10.1093/jxb/eru205 (PMC4085968; doi:10.1093/jxb/eru205)
Supplement: Supplementary Data [file supp_eru205_jexbot118547_file001.pdf]

Increasing water use efficiency along the C3-to-C4 evolutionary pathway: a stomatal optimization perspective

Danielle A Way, Gabriel G Katul, Stefano Manzoni, and Giulia Vico

Table S1 – Parameter table

**Species**	**Photosynthetic type**	**Rubisco site concentration (µmol m^{-2})**	**k_{cat} (mol mol^{-1} s^{-1})**	**$V_{c,max}$ (µmol m^{-2} s^{-1})**	**K_c (µM)**	**K_o (µM)**	**Γ (µmol mol^{-1})**	**CE**
		D. Kubien (pers. comm.)	Kubien et al. 2008	Calculated	Kubien et al. 2008	Kubien et al. 2008	Ku et al. 1991	Sudderth et al. 2007, citing Dai et al. 1996 or *Krall et al. 1991*
F. cronquistii	C3				10.8	653	60.4	0.14
F. pringlei	C3	17.1	3.11	53.18	12	666	62	0.13, *0.071*
F. angustifolia	Type 1	25.6	2.86	73.22	13.1		24.1	
F. chlorofolia	Type 1	19.7	3.35	66.00	12.4	740	29	
F. sonorensis	Type 1	28	2.69	75.32	10.2	785	29.6	*0.079*
F. pubescens	Type 1						21.3	
F. floridana	Type 2	28.3	3.19	90.28	13.2	686	9.5	*0.095*
F. ramossissima	Type 2	18.4	2.77	50.97	12	722	9	0.17
F. brownii	Type 3	12.2	2.58	31.48	12.8	378	6	0.16, *0.143*
F. palmeri	Type 3	10.8	3.54	38.23	13.5	193	4.7	0.38
F. vaginata	Type 3	9.3	3.78	35.15	21.4	880	3	0.4
F. australasica	C4	11.4	3.84	43.78	22	309		0.46
F. bidentis	C4	10.9	4.16	45.34	20.2	639	3.2	0.44, *0.476*
F. kochiana	C4	10.8	3.68	39.74	22.7	150	2.2	
F. trinervia	C4	6.5	4.42	28.73	17.9	671	3.5	0.52
